# Correlation between Circulating miR-16, miR-29a, miR-144 and miR-150, and the Radiotherapy Response and Survival of Non-Small-Cell Lung Cancer Patients

**DOI:** 10.3390/ijms241612835

**Published:** 2023-08-16

**Authors:** Matthias Bache, Frauke Kadler, Olivia Struck, Daniel Medenwald, Christian Ostheimer, Antje Güttler, Jacqueline Keßler, Matthias Kappler, Anne Riemann, Oliver Thews, Barbara Seliger, Dirk Vordermark

**Affiliations:** 1 Department of Radiotherapy, Martin Luther University Halle-Wittenberg, Ernst-Grube-Str. 40, 06120 Halle, Germany; frauke.kadler@uk-halle.de (F.K.); olivia.struck@uk-halle.de (O.S.); daniel.medenwald@uk-halle.de (D.M.); antje.guettler@uk-halle.de (A.G.); jacqueline.kessler@uk-halle.de (J.K.); dirk.vordermark@uk-halle.de (D.V.); 2 Department of Radiology, Martin Luther University Halle-Wittenberg, Ernst-Grube-Str. 40, 06120 Halle, Germany; 3 Department of Oral and Maxillofacial Plastic Surgery, Martin Luther University Halle-Wittenberg, Ernst-Grube-Str. 40, 06120 Halle, Germany; matthias.kappler@uk-halle.de; 4 Julius Bernstein Institute of Physiology, Martin Luther University Halle-Wittenberg, Magdeburger Str. 6, 06112 Halle, Germany; anne.riemann@medizin.uni-halle.de (A.R.); oliver.thews@medizin.uni-halle.de (O.T.); 5 Medical Faculty, Martin Luther University Halle-Wittenberg, Magdeburger Str. 16, 06112 Halle, Germany; barbara.seliger@uk-halle.de; 6 Institute for Translational Immunology, Brandenburg Medical School “Theodor Fontane”, 14770 Brandenburg, Germany; 7 Fraunhofer Institute for Cell Therapy and Immunology, 04103 Leipzig, Germany

**Keywords:** non-small-cell lung cancer, circulating microRNAs, radiotherapy, prognosis, digital droplet PCR

## Abstract

Despite the success of current therapy concepts, patients with advanced non-small-cell lung cancer (NSCLC) still have a very poor prognosis. Therefore, biological markers are urgently needed, which allow the assessment of prognosis, or prediction of the success of therapy or resistance in this disease. Circulating microRNAs (miRs) have potential as biomarkers for the prognosis and prediction of response to therapy in cancer patients. Based on recent evidence that circulating miR-16, miR-29a, miR-144 and miR-150 can be regulated by ionizing radiation, the concentration of these four miRs was assessed in the plasma of NSCLC patients at different time points of radiotherapy by digital droplet PCR (ddPCR). Furthermore, their impact on patients’ prognosis was evaluated. The mean plasma levels of miR-16, miR-29a, miR-144 and miR-150 significantly differed intra- and inter-individually, and during therapy in NSCLC patients, but showed a strong positive correlation. The individual plasma levels of miR-16, miR-29a and miR-144 had prognostic value in NSCLC patients during or at the end of radiotherapy in Cox’s regression models. NSCLC patients with low levels of these three miRs at the end of radiotherapy had the worst prognosis. However, miR-150 plasma levels and treatment-dependent changes were not predictive. In conclusion, circulating miR-16, miR-29a and miR-144, but not miR-150, have a prognostic value in NSCLC patients undergoing radiotherapy.

## 1. Introduction

Lung cancer is one of the leading causes of cancer death worldwide [1]. The survival rate for non-small-cell lung carcinoma (NSCLC) depends on various factors, such as the subtype of lung cancer and the stage of disease, with a 5-year survival rate of approximately 28% (https://www.cancer.net/cancer-types/lung-cancer-non-small-cell/statistics (accessed on 1 February 2023)). For the treatment of advanced lung cancer, multimodal therapy concepts are used, including surgery, radiation and chemotherapy. In the case of inoperable lung cancer, the current standard therapy is radiotherapy combined with chemotherapy. Although both therapies have undergone further development in the last decades and immunotherapy has become a new option for NSCLC patients, a partial response and acquisition of resistance prevents treatment success, and this disease is still associated with a very poor patient prognosis (Chen et al., 2020, Lafaze et al., 2023, Zhou et al., 2023 [2,3,4,5,6,7,8]).

Molecular and cellular markers provide additional information regarding the prognosis and prediction of the success of or resistance to treatment, which allows an estimation of the benefit of therapy for NSCLC. In this context, the National Comprehensive Cancer Network (NCCN) has recently recommended comprehensive testing of numerous biomarkers such as ALK, BRAF, EGFR, KRAS, METex14 skipping, NTRK1/2/3, RET, ROS1, high-grade MET amplification, ERBB2 mutations and PD-L1 in patients with NSCLC [9]. In particular, liquid biopsies have emerged as a promising tool for the detection of biomarkers in this disease, and they are valuable for early intervention and personalized treatment and might improve patient outcomes [10,11]. Liquid biopsies include the patient’s blood, urine or other body fluids for the detection of biomarkers. An advantage of liquid biopsies is that they are also minimally invasive compared to traditional tissue biopsies and are thus more convenient and less risky for patients. Liquid biopsies allow for the analysis of circulating tumor DNA (ctDNA), circulating tumor cells (CTCs), extracellular vesicles (EVs) and microRNAs (miRs). For example, the composition of the immune cell subpopulations, different blood markers and their combinations, such as cytokeratin-19 fragment (Cyfra 21-1), carcino-embryonic antigen (CEA) and neuron-specific enolase (NSE), has a prognostic value for lung cancer [12,13]. In addition, our own studies have identified hypoxic tumor markers, such as osteopontin (OPN), vascular endothelial growth factor (VEGF) and carbonic anhydrase IX (CAIX) as prognostic factors in NSCLC with radiation therapy (RT) [14,15]. 

Over the last decade, the expression, regulation and function, as well as the clinical relevance of miRs have been intensively studied in solid cancers, including NSCLC, which gives further insights into the process of neoplastic transformation, metastases formation and treatment efficacy. MiRs are small non-coding RNAs that mainly bind to the 3′untranslated region (3′UTR) of mRNA molecules and regulate the post-transcriptional gene expression of, e.g., oncogenes, tumor suppressors and other key proteins [16]. Numerous circulating miRs have been identified as prognostic and predictive factors for NSCLC [17,18,19]. However, the data are not consistent, and sometimes even conflicting, depending on methodological variables [20]. Therefore, it is strongly recommended that miRs should be validated in large cohorts and independent studies before they can be proposed as biomarkers for clinical use [21,22].

In a previous report, miR-29a, miR-150, miR-144 and miR-16 were shown to be regulated by ionizing radiation. Consequently, they might serve as relevant biomarkers for radiotherapy. Furthermore, miR-29a and miR-150 were identified in NSCLC patients as circulating biomarkers that correlated with the delivered RT dose, with an increase in intracellular levels of miR-29a and miR-150 in NSCLC cells after irradiation, but a decrease in exosomes [23]. MiR-29a influenced the expression of numerous oncogenes and tumor-suppressive activity in solid tumors [24]. In contrast, the role of miR-150 in tumor progression or tumor suppression is unclear as both oncogenic and tumor-suppressive effects have been described for this miR in solid tumors [25]. Several studies have shown that miR-144 affects proliferation, apoptosis and radiosensitivity in tumor cell lines [26,27,28]. In a meta-analysis, miR-144 was downregulated in NSCLC and correlated with stage, lymph node metastasis and vascular invasion [29]. Upregulation of miR-144 was also detected in blood samples from rats, 2 weeks after lung irradiation [30]. Generally, miR-144 acts as a tumor suppressor by regulating the gene expression of numerous key cell proteins and oncogenes [31]. MiR-16 induced apoptosis and was downregulated in radioresistant lung cancer cells [32]. A meta-analysis showed that low expression levels of miR-16 were associated with a poor prognosis in solid cancer patients [33]. In general, miR-16 was described as an miR with tumor suppressor functions, was involved in the regulation of MHC molecules, and was downregulated in blood samples from various tumor entities and in tumor cell lines [34,35,36]. To determine the prognostic value of these four selected circulating miRs, the plasma levels of the miRs were measured in the blood of 178 patients with locally irresectable NSCLC using digital droplet PCR (ddPCR). Moreover, we correlated the miR levels at different time points of radiotherapy (prior, during and at the end of radiotherapy) with each other and with overall survival (OS).

## 2. Results

### 2.1. miR Expression Levels—Correlation Coefficients and Influencing Factors

**Figure 1:**
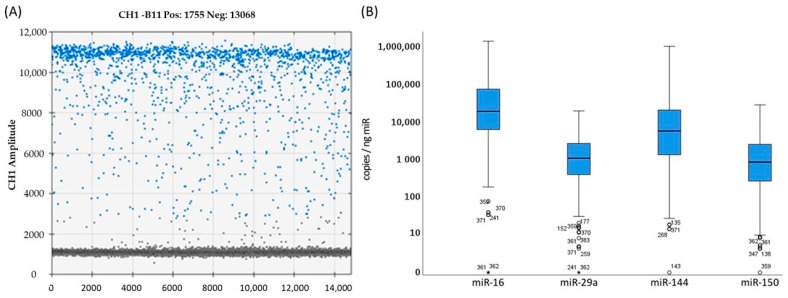
(**A**) Example of separation of positive and negative droplets of miR-150 of a patient sample using ddPCR. (**B**) Distribution of expression levels of miR-16, miR-29a, miR-144 and miR-150 in copies/ng total miRNA in 455 samples from 178 NSCLC patients. Asterisks and circles indicate the miR expression level of single patients.

The expression levels of miR-16, miR-29a, miR-144 and miR-150 were analyzed in 455 plasma samples from 178 NSCLC patients (Table 1, Figure 1). 

Among the four miRNAs, miR-16 exhibited the highest median expression level, with 19,609 copies/ng total miR. In comparison, miR-29a, miR-144 and miR-150 displayed lower expression levels, with values of 1111 copies/ng total miR, 5888 copies/ng total miR and 872 copies/ng total miR, respectively (Table 1, Figure 1). Specifically, miR-29a, miR-144 and miR-150 were approximately 19-fold-, 3-fold- and 22-fold-less expressed than miR-16 in NSCLC patients. The miRNA expression levels (miR-16, miR-29a, miR-144 and miR-150) exhibited a strong correlation with each other, as evidenced by correlation coefficients (r_s_) ranging from 0.79 to 0.90 (*p* < 0.001) (Table 2).

**Figure 2:**
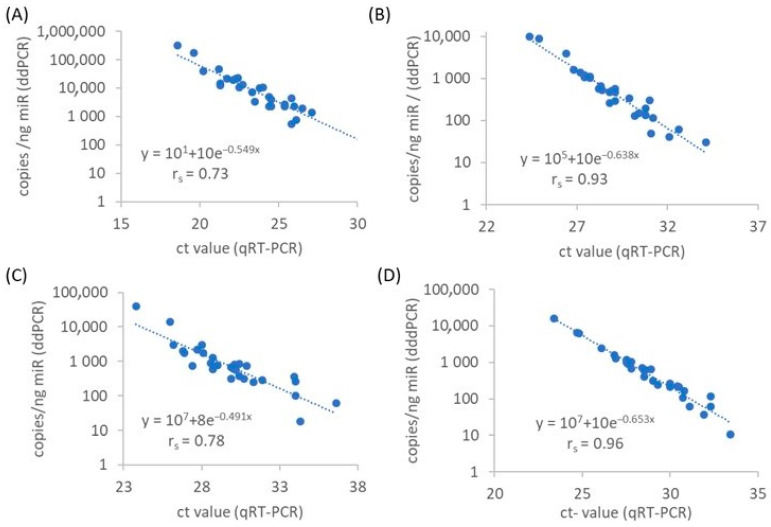
Correlation analysis of miR-16 (**A**), miR-29a (**B**), miR-144 (**C**) and miR-150 (**D**) plasma levels measured by ddPCR and qRT-PCR in NSCLC patients (n = 30) according to Spearman’s Rho (r_s_).

To assess the relationship between the two different quantification methods, ddPCR and quantitative real-time PCR (qRT-PCR), a correlation coefficient was determined. The results indicated a correlation between the miRNA concentrations measured by ddPCR and qRT-PCR for the four examined miRs, with correlation coefficient (r_s_) values ranging from 0.73 to 0.96 (Figure 2).

In order to address possible bias factors, several factors were examined, including free plasma hemoglobin (Hb) levels, total miR concentration and duration of sample storage. The plasma samples were collected over a period of three years, with a median storage time of 1.6 years and a range of 0.1 to 3.4 years. The Hb value of the study samples had a median of 0.027 g/L, ranging from 0.0001 to 1.08 g/L, indicating no or low levels of hemolysis. The median total miR concentration was 8.0 ng/μL, with a range of 0.6 to 101.3 ng/μL. The correlation coefficients (r_s_) between hemolysis and miR expression levels or total miR concentrations and miR expression levels were ≤0.26, suggesting a minor effect on the analyzed miRs. The correlation coefficients (r_s_) between miR expression levels and the age of the samples ranged from −0.28 to −0.45 (Table 3). These findings suggest that these bias parameters should be checked and might be taken into account in the analysis due to their potential impact on the analyzed miRNAs, in order to ensure the validity and reliability of the results.

### 2.2. miR-16, miR-29a, miR-144 and miR-150 Plasma Levels Prior to, during and Post-Radiotherapy Treatment

**Figure 3:**
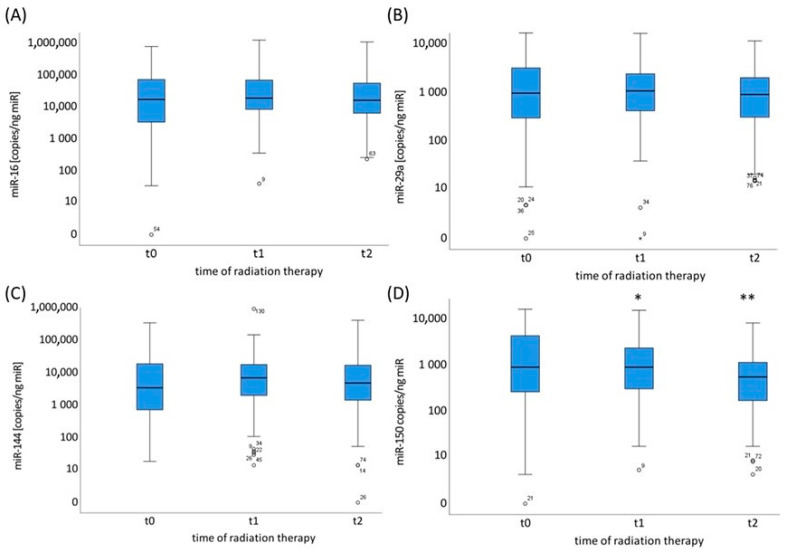
Expression level distribution of miR-16 (**A**), miR-29a (**B**), miR-144 (**C**) and miR-150 (**D**) in copies/ng total miR in the plasma of NSCLC patients (n = 110) before radiotherapy (t0), at a radiation dose of 20 Gy (t1) and at the end of radiotherapy (t2), (*p* < 0.05 *, *p* < 0.001 **). Circles indicate the miR expression level of single patients.

The plasma levels of miR-16, miR-29a, miR-144 and miR-150 in NSCLC patients (n = 110) were analyzed over the course of radiotherapy—before radiotherapy (t0), at a radiation dose of 20 Gy (t1), and at the end of radiotherapy (t2). The results showed that only miR-150 exhibited a significant decrease in expression levels after 20 Gy (t1) to 82% (*p* = 0.04), which was further pronounced at the end of radiotherapy (t2) to 58% (*p* < 0.001) (Figure 3D). In contrast, the expression levels of miR-16, miR-29a and miR-144 showed no significant changes compared to the levels prior to radiotherapy (Figure 3A–C).

### 2.3. miR-16, miR-29a, miR-144 and miR-150 Plasma Levels Prior to, during and Post-Radiotherapy Treatment

For the OS analysis, blood samples from NSCLC patients were analyzed at different time points of radiotherapy. A total of 175, 128 and 144 patients were included prior to RT (t0), after 20 Gy (t1) and at the end of radiotherapy (t2), respectively. The effects of circulating miRs at different time points of radiotherapy (t0, t1, t2) on OS were assessed using univariate and multivariate Cox’s regression models adjusted for age, sex and UICC stage. The median expression levels of miR-16, miR-29a, miR-144 and miR-150 were used to evaluate their association with OS (Table 4).

The results showed that the pretherapeutic expression levels of miR-16, miR-29a, miR-144 and miR-150 had no significant association with OS (Table 5).

However, in the univariate Cox’s regression models, a low level of miR-29a at t1 and a low miR-144 level at t2 were significantly associated with an increased risk of death (RR = 1.6, *p* = 0.04 and RR = 1.6, *p* = 0.03) (Table 6). In addition, we observed a trend suggesting an association of low miR-16 and miR-29a levels at t2 with an increased risk of death (RR = 1.5, *p* = 0.07 and RR = 1.5, *p* = 0.08) in univariate Cox’s regression models. Furthermore, in the multivariate Cox’s regression model adjusted for age, sex and UICC stage, a low miR-144 level was also associated with an increased risk of death (RR = 1.5, *p* = 0.06).

**Figure 4:**
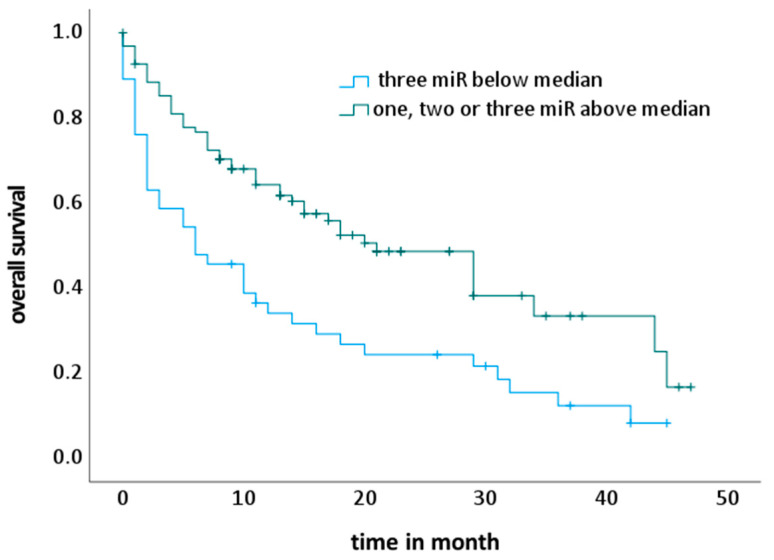
OS of NSCLC patients in Kaplan–Meier analysis: association of combined plasma levels of miR-16, miR-29a and miR-144 at the end of radiotherapy (t2).

Kaplan–Meier analyses revealed that of the 144 NSCLC patients at the end of radiotherapy (t2), 46 patients with low levels of all three miRs (miR-16, miR-29a, miR-144) had a significant reduction in OS compared with the other patients (*p* < 0.001; log-rank test) (Figure 4). The relative risk (RR) of this patient group with the worst prognosis was calculated as 2.0 (CI: 1.3–3.1, *p* = 0.003) compared to the control group for multivariate analysis (Table 7). However, no significant associations between miR-150 and changes in miR-150 expression levels were observed to affect the OS of NSCLC patients.

## 3. Discussion

The use of circulating miRs as biomarkers for detection, prognosis and prediction in lung cancer has been investigated in several studies [17,18,19]. In this study, we focused on evaluating the significance of four specific miRs (miR-16, miR-29a, miR-144 and miR-150) in NSCLC patients, which have been demonstrated to be important in the context of radiotherapy both in vitro and in vivo. For example, miR-16 has been found to induce apoptosis and is downregulated by the WEE1 G2 checkpoint kinase in radioresistant lung cancer cells [32]. A study has also shown a decrease in miR-29a and miR-150 expression levels with increasing radiation dose in NSCLC patients [23]. Additionally, miR-144 has been found to inhibit the proto-oncogene MET, leading to proliferation arrest, apoptosis and increased radiosensitivity in tumor cells [26,27,28]. However, it should be noted that considerable variation and conflicting data exist, particularly in the field of circulating miRs, which requires further review and evaluation of their impact in independent studies. Our study contributes to the existing body of knowledge by investigating the significance of the expression levels of these specific miRs in NSCLC patients undergoing radiotherapy at different time points (prior to radiotherapy, after 20 Gy and at the end of radiotherapy). A meta-analysis showed considerable heterogeneity in irradiation doses for miR analysis between different studies [37]. Dinh et al. identified the circulating miR-29a and miR-150 during radiation therapy for non-small-cell lung cancer after 20 and 40 Gy [23]. The quantification of miRs using qRT-PCR is widely used but is subject to certain limitations. One of the main challenges is the difficulty in achieving standardized quantification across different studies due to the use of various controls for normalization. This lack of consistency and agreement among different cancer studies has been highlighted in meta-analyses of circulating miR studies [38,39,40]. Consequently, there is still an ongoing search for a universally accepted and standardized quantification method using qRT-PCR.

To address this challenge, ddPCR has emerged as an alternative method that enables absolute quantification of the number of miR copies without the need for reference miRs. In serum samples, the use of ddPCR has demonstrated improved precision, reducing measurement scatter for miR-141 by a factor of seven compared to qRT-PCR [41]. Additionally, a study conducted by Campomenosi et al. found a strong correlation between qRT-PCR and ddPCR for various miRs in the serum from NSCLC patients [42]. Notably, ddPCR exhibited less variability in repeat measurements compared to qRT-PCR [42]. Our investigations have provided evidence of a significant positive correlation between qRT-PCR and ddPCR (Figure 2). However, the values of the correlation coefficients ranged from 0.73 to 0.96, demonstrating a significant difference among the four miRs. Further studies of other miRs, e.g., the systemic review carried out by Malachowska and coauthors [37], are required to underline the strong correlation between circulating miRs and radiation overall. Nevertheless, the determination of the absolute number of miR copies provides the advantage that absolute cut-off values for risk assessment can be defined, which are independent from the individual lab techniques (e.g., selection of the housekeeper gene). In addition, further studies have demonstrated that ddPCR has the potential to identify miRs with diagnostic and prognostic value [43]. Overall, these results suggest that ddPCR is an excellent alternative to qRT-PCR for identifying circulating miRs with prognostic significance.

The mean expression levels of miR-16, miR-29a, miR-144 and miR-150 exhibited significant differences from each other (Figure 1B). However, despite these differences, there was a strong positive correlation observed among these miRs in NSCLC patients (Table 2). This correlation suggests the possibility of a similar functional role for these miRs as tumor suppressors in NSCLC. Indeed, miR-16 downregulation has been reported in various tumor types, highlighting its potential tumor suppressor role across different cancer entities [36]. In the context of NSCLC, studies have demonstrated that miR-16 can induce apoptosis and inhibit the proliferation and migration of NSCLC cells in vitro [44,45]. However, circulating miR-16 has also been used as a reference miR in various studies, including NSCLC patient studies.

Regarding miR-29a, its downregulation in NSCLC patients has been observed in multiple studies, highlighting its potential tumor-suppressive role [46,47]. Additionally, miR-29a has been shown to reduce the activity of the Wnt/β-catenin signaling pathway in NSCLC cells, which is associated with tumor growth and progression [48]. MiR-144 has been reported to be downregulated in various tumor entities and has functions as a tumor suppressor by inhibiting cell proliferation, invasion, metastasis and tumor growth [29,49,50]. MiR-150 has been shown to play a role in inhibiting cancer stem-cell-induced tumorigenesis, recurrence and metastasis in NSCLC [51].

In our analysis, only the miR-150 plasma levels of all NSCLC patients were associated with radiotherapy, and decreased significantly during and at the end of radiotherapy (Figure 3). Consistent with our results, miR-150 was one of four miRs identified in a meta-analysis by the authors in [37] that correlated significantly with radiation exposure. However, the decrease or increase in miR-150 levels due the RT does not have any prognostic value for NSCLC patients. Further studies on different cohorts with larger patient numbers are required to verify these data.

Consistent with their proposed functional role as tumor suppressors, low plasma levels of miR-16, miR-29a and miR-144 during or at the end of radiotherapy significantly correlated or showed a trend of association with an increased risk of death in a univariate Cox’s regression model (Table 6). Furthermore, NSCLC patients with low plasma levels of these three miRs at the end of radiotherapy exhibited the highest risk of death in the multivariate Cox’s regression analysis (Table 7). The OS of patients with a low level of these three miRs at the end of radiotherapy was significantly decreased (*p* < 0.001) in the Kaplan–Meier analysis (Figure 4). The use of tumor-suppressive miRNA mimics or a reduction in oncogenic miRNAs may be a promising new therapeutic strategy to overcome resistance to radiation and drugs caused by driver mutations in cancer patients, including those with NSCLC [52,53,54].

Taken together with the results of previous studies, our study successfully demonstrated the importance of selected miRs for radiotherapy. The selected miRs (except for miR-150) exhibit predictive value at the end of radiotherapy and can identify patients who may benefit from additional therapy. Several more recent reviews [19,55,56,57,58] have listed numerous other miRs that are important for radiation and chemoresistance in NSCLC. However, the prognostic and predictive value of these miRs needs to be reassessed in further patient studies.

## 4. Materials and Methods

### 4.1. Patients, Treatment Blood Samples, Hemoglobin (Hb) and Plasma Isolation

From July 2017 to October 2020, a total of 178 patients with local, inoperable NSCLC who were undergoing radiotherapy were prospectively enrolled in this study. The patient characteristics and clinical data are presented in Table 8. The inclusion criteria for study participation were a histologically confirmed NSCLC, age of ≥18 years and no history of other cancers in the last five years.

For plasma isolation, blood samples were collected from patients with NSCLC using EDTA plasma tubes (Sarstaedt, Nümbrecht, Germany) at different time points of RT. The blood samples were centrifuged at room temperature for 10 min at 2000× *g*, and the supernatant was collected and stored at −80 °C. Plasma samples were examined from all patients (n = 178) before radiotherapy (t0), from 130 patients who received a radiation dose of 20 Gy (t1) and from 147 patients at the end of radiotherapy (t2).

To assess the degree of hemolysis in the blood samples, absorbance was measured at 415 nm, 380 nm and 450 nm using a Nanodrop 2000c spectrophotometer (Thermo Fisher Scientific, Dreiech, Germany). The concentration of free hemoglobin (Hb) was determined using the 3-wavelength method according to Harboe, using the following formula [59]:

Hb [g/L] = (167.2 × A415 − 83.6 × A380 − 83.6 × A450) × 0.01017

### 4.2. Total miR Isolation, cDNA Synthesis, qRT-PCR and ddPCR

Total miR was isolated from 250 µL EDTA plasma using the miRNeasy serum/plasma Advanced Kit (Qiagen, Hilden, Germany), following the manufacturer’s instructions. The concentration of total miR was analyzed using a NanoDrop 2000c spectrophotometer (Thermo Fisher Scientific). A concentration of 1 ng/µL of total miR was used for the cDNA synthesis, which was performed using the miRCURY LNA RT Kit (Qiagen, Hilden, Germany) according to the manufacturer’s instructions. The resulting cDNA was stored at −20 °C.

For qRT-PCR, the 1:30 diluted cDNA was added to the 2× miRCURY SYBR Green Master Mix, along with the miRCURY LNA miRNA PCR assay primers (refer to Table 9), following the manufacturer’s instructions (Qiagen). The qRT-PCR reaction was conducted using a Rotor-Gene 6000 (Qiagen), as previously described [60]. The total miR concentration was used for normalization by qRT-PCR.

The ddPCR was determined according to manufacturer’s protocols. The cDNA was added to ddPCR EvaGreen Supermix (Bio-Rad Laboratories, Feldkirchen, Germany) along with the miRCURY LNA miRNA PCR assay primers (refer to Table 9). The cDNAs were diluted as follows: 1:25 for miR-29a and miR-150; 1:100 for miR-144; and 1:250 for miR-16. The measurement of positive and negative droplets was carried out using the QX200 ™ Droplet Reader (Bio-Rad Laboratories). The absolute copy count was calculated based on the droplet count using the Poisson distribution and the QuantaSoft software (Bio-Rad Laboratories).

### 4.3. Statistics

All statistical analyses were conducted using the IBM SPSS software package, version 28.0. Differences in numerical data between groups were assessed using the Mann–Whitney *U* test or Kruskal–Wallis test. Spearman’s correlation coefficient was used to measure the strength and direction of association between the two ranked variables. Survival curves were generated using Kaplan–Meier analysis and the log-rank test was applied to assess differences. For univariate analyses and multivariate analyses (adjusted for age, sex and UICC stage), the Cox’s proportional hazard model was utilized to calculate the hazard ratio in the analysis of OS. A total of 175, 128 and 142 patients were included for survival analysis before radiotherapy (t0), after 20 Gy (t1) and at the end of radiotherapy (t2), respectively. A p-value of less than 0.05 was considered to be statistically significant.

## 5. Conclusions

The present study suggests that miR-16, miR-29a and miR-144, but not miR-150, have prognostic value for patients with NSCLC, during or at the end of radiotherapy. The combination of different miRs enhances the predictive power. Since reassessment of the radiobiological relevance is crucial for utilizing circulating miRs as clinical biomarkers, the impact of these miRs should be assessed in larger, independent cohorts.

## Figures and Tables

**Table 1:** Median and mean expression levels of miR-16, miR-29a, miR-144 and miR-150 in 455 samples from 178 NSCLC patients measured by ddPCR.

	Expression Levels [Copies/ng Total miR]			
	miR-16	miR29a	miR-144	miR-150
Median	19,609	1111	5888	872
Mean	71,674	2239	22,620	2210

**Table 2:** Correlation coefficients (r_s_) between miR-16, miR-29a, miR-144 and miR-150 plasma levels in NSCLC patients (n = 455) described by Spearman’s Rho (r_s_).

	miR-29a	miR-144	miR-150
miR-16	0.82 **	0.83 **	0.81 **
miR-29a		0.85 **	0.90 **
miR-144			0.79 **

*p* < 0.001 **.

**Table 3:** Correlation coefficients (r_s_) between miR-16, miR-29a, miR-144 and miR-150 plasma levels and hemolysis, total miR concentration or duration of sample storage in NSCLC patients samples (n = 455) according to Spearman’s Rho (r_s_).

Parameter	miR-16	miR-29a	miR-144	miR-150
Hemolysis	0.25 **	0.10 *	0.26 **	0.11 *
miR-concentration	−0.24 **	−0.23 **	−0.26 **	−0.25 **
Duration of sample storage	−0.34 **	−0.32 **	−0.45 **	−0.28 **

*p* < 0.05 *, *p* < 0.001 **.

**Table 4:** Median expression levels of miR-16, miR-29a, miR-144 and miR-150 prior to, during and post radiotherapy.

		Median Expression Levels [Copies/ng Total miR]			
RT	No. of Cases	miR-16	miR-29a	miR-144	miR-150
t0	175	22,053	1139	5044	1289
t1	128	19,442	1103	7066	917
t2	144	18,109	1164	6210	694

Abbreviations: RT—time point of radiotherapy, No. of cases—number of cases, t0—prior to irradiation, t1—after irradiation with 20 Gy, t2—at the end of irradiation.

**Table 5:** Univariate Cox’s regression analysis for the association of miR-16, miR-29a, miR-144 or miR-150 level and overall survival of NSCLC patients prior to radiotherapy.

			Univariate Analysis		
miR	Median	Subject Subgroup	RR	95% CI	*p*-Value
miR-16	</≥	<median	1.1	0.78–1.68	0.48
miR- 29a	</≥	<median	1.1	0.78–1.68	0.49
miR-144	</≥	<median	1.1	0.76–1.65	0.57
miR-150	</≥	<median	1.2	0.84–1.82	0.28

Abbreviations: RR—relative risk, CI—confidence interval.

**Table 6:** Univariate and multivariate Cox’s regression analysis for the association of miR-16, miR-29a or miR-144 level and OS of NSCLC patients during and post-radiotherapy.

				Univariate Analysis			Multivariate Analysis		
miR	Median	Subject Subgroup	RT	RR	95% CI	*p*-Value	RR	95% CI	*p*-Value
miR-29a	</≥	<median	t1	1.6	1.02–2.46	0.04	1.3	0.84–2.06	0.23
miR-16	</≥	<median	t2	1.5	0.97–2.27	0.07	1.4	0.92–2.17	0.11
miR-29a	</≥	<median	t2	1.5	0.96–2.22	0.08	1.4	0.94–2.18	0.10
miR-144	</≥	<median	t2	1.6	1.05–2.49	0.03	1.5	0.98–2.35	0.06

Abbreviations: RT—time point of radiotherapy, RR—relative risk, CI—confidence interval, t1—at a radiation dose of 20 Gy and t2—at the end of radiotherapy.

**Table 7:** Multivariate Cox’s regression analysis (adjusted for age, sex and UICC stage) for the association of a combination of miR-16, miR-29a and miR-144 scores at the end of radiotherapy (t2) and OS of NSCLC patients.

			Multivariate Analysis		
miRs	Groups	Subject Subgroup	RR	95% CI	*p*-Value
miR-16, miR-29a	Both miRs below median vs. others	Both miRs below median	1.7	1.1–2.6	0.02
miR-16, miR-144	Both miRs below median vs. others	Both miRs below median	1.6	1.0–2.5	0.03
miR-29a, miR-144	Both miRs below median vs. others	Both miRs below median	1.5	0.9–2.1	0.19
miR-16, miR-29a, miR-144	All three miRs below median vs. others	All three miR below median	2.0	1.3–3.1	0.003

Abbreviations: RR—relative risk, CI—confidence interval.

**Table 8:** Patient and treatment characteristics of 178 NSCLC patients.

Characteristics		Number of Cases (%)
Sex	Female	53 (30%)
	Male	125 (70%)
Age	Median	69 (range 45–90)
Histology	Adeno-Ca	75 (42%)
	SCC	68 (38%)
	Others	35 (20%)
Localization	Central	38 (21%)
	Peripheral	138 (74%)
	Unknown	9 (5%)
Differentiation	Well–moderate	40 (23%)
	Poor	50 (28%)
	Undifferentiated	1 (0%)
	Unknown	87 (49%)
UICC stage	I	20 (11%)
	II	14 (8%)
	III	61 (34%)
	IV	73 (41%)
	Unknown	10 (6%)
GTV	Median	27.9 mL (range 1.5–1597 mL)
Karnofsky Index	Median	80 (range 50–100)
Smoking status	No	12 (7%)
	es	112 (63%)
	Unknown	54 (30%)
Radiotherapy	Mean total dose	49 Gy (range 6–83 Gy)
	+Chemotherapy	84 (48%)
	+Immunotherapy	55 (31%)
	+Chemotherapy + immunotherapy	35 (20%)
Stereotactic radiotherapy	Yes	52 (29%)
	No	126 (71%)

Abbreviations: Adeno-Ca—adenocarcinoma, SCC—squamous cell carcinoma, GTV—gross tumor volume.

**Table 9:** Primer order number with name and sequence of the associated miR.

Order Number	miR	Sequence
YP00205702	hsa-miR-16-5p (miR-16)	5′-UAGCAGCACGUAAAUAUUGGCG-3′
YP00204698	hsa-miR-29a-3p (miR-29a)	5′-UAGCACCAUCUGAAAUCGGUUA-3′
YP00204754	hsa-miR-144-3p (miR-144)	5′-UACAGUAUAGAUGAUGUACU-3′
YP00204660	hsa-miR-150-5p (miR-150)	5′-UCUCCCAACCCUUGUACCAGUG-3′

## Data Availability

The data presented in this study are available on request from the corresponding author.

## Abbreviations

Carbonic anhydrase IX (CAIX); circulating tumor cells (CTCs); circulating tumor DNA (ctDNA); digital droplet PCR (ddPCR); extracellular vesicles (EVs); hemoglobin (Hb); microRNA(s) (miR(s)); non-small-cell lung cancer (NSCLC); osteopontin (OPN); overall survival (OS); quantitative real-time PCR (qRT-PCR); radiation therapy (RT); vascular endothelial growth factor (VEGF); 3′untranslated region (3′UTR).

## References

[1] World Health Organization Cancer. https://www.who.int/news-room/fact-sheets/detail/lung-cancer.

[2] Zhou S., Yang H. (2023). Immunotherapy resistance in non-small-cell lung cancer: From mechanism to clinical strategies. Front. Immunol..

[3] Laface C., Maselli F.M., Santoro A.N., Iaia M.L., Ambrogio F., Laterza M., Guarini C., de Santis P., Perrone M., Fedele P. (2023). The Resistance to EGFR-TKIs in Non-Small Cell Lung Cancer: From Molecular Mechanisms to Clinical Application of New Therapeutic Strategies. Pharmaceutics.

[4] Chen R., Manochakian R., James L., Azzouqa A.-G., Shi H., Zhang Y., Zhao Y., Zhou K., Lou Y. (2020). Emerging therapeutic agents for advanced non-small cell lung cancer. J. Hematol. Oncol..

[5] Huber R.M., de Ruysscher D., Hoffmann H., Reu S., Tufman A. (2019). Interdisciplinary multimodality management of stage III nonsmall cell lung cancer. Eur. Respir. Rev..

[6] Król K., Mazur A., Stachyra-Strawa P., Grzybowska-Szatkowska L. (2023). Non-Small Cell Lung Cancer Treatment with Molecularly Targeted Therapy and Concurrent Radiotherapy—A Review. Int. J. Mol. Sci..

[7] Liu L., Yuan M., Ding Z., Feng H., Zhang X., Liang N., Wang W., Wang J., Zhang Y., Zhou S. (2022). Immunotherapy with radiotherapy fails to improve prognosis of patients with stage IV non-small cell lung cancer: A retrospective cohort analysis of the THUNDER-2 study. Transl. Lung Cancer Res..

[8] Zhang X., Zhang J., Liu P., Wang J., Zhao K., Zhu Z., Gu K., Zhao W. (2023). Immunotherapy progress and clinical strategy of unresectable locally advanced non-small cell lung cancer. Front. Oncol..

[9] Ettinger D.S., Wood D.E., Aisner D.L., Akerley W., Bauman J.R., Bharat A., Bruno D.S., Chang J.Y., Chirieac L.R., D’Amico T.A. (2022). Non-Small Cell Lung Cancer, Version 3.2022, NCCN Clinical Practice Guidelines in Oncology. J. Natl. Compr. Canc. Netw..

[10] Souza V.G.P., Forder A., Brockley L.J., Pewarchuk M.E., Telkar N., de Araújo R.P., Trejo J., Benard K., Seneda A.L., Minutentag I.W. (2023). Liquid Biopsy in Lung Cancer: Biomarkers for the Management of Recurrence and Metastasis. Int. J. Mol. Sci..

[11] Casagrande G.M.S., Silva M.d.O., Reis R.M., Leal L.F. (2023). Liquid Biopsy for Lung Cancer: Up-to-Date and Perspectives for Screening Programs. Int. J. Mol. Sci..

[12] Barlési F., Gimenez C., Torre J.-P., Doddoli C., Mancini J., Greillier L., Roux F., Kleisbauer J.-P. (2004). Prognostic value of combination of Cyfra 21-1, CEA and NSE in patients with advanced non-small cell lung cancer. Respir. Med..

[13] Jiang C., Zhao M., Hou S., Hu X., Huang J., Wang H., Ren C., Pan X., Zhang T., Wu S. (2022). The Indicative Value of Serum Tumor Markers for Metastasis and Stage of Non-Small Cell Lung Cancer. Cancers.

[14] Ostheimer C., Bache M., Güttler A., Kotzsch M., Vordermark D. (2014). A pilot study on potential plasma hypoxia markers in the radiotherapy of non-small cell lung cancer. Osteopontin, carbonic anhydrase IX and vascular endothelial growth factor. Strahlenther. Onkol..

[15] Ostheimer C., Bache M., Güttler A., Reese T., Vordermark D. (2014). Prognostic information of serial plasma osteopontin measurement in radiotherapy of non-small-cell lung cancer. BMC Cancer.

[16] O’Brien J., Hayder H., Zayed Y., Peng C. (2018). Overview of MicroRNA Biogenesis, Mechanisms of Actions, and Circulation. Front. Endocrinol..

[17] Inamura K. (2017). Major Tumor Suppressor and Oncogenic Non-Coding RNAs: Clinical Relevance in Lung Cancer. Cells.

[18] Lampignano R., Kloten V., Krahn T., Schlange T. (2020). Integrating circulating miRNA analysis in the clinical management of lung cancer: Present or future?. Mol. Aspects Med..

[19] Zhong S., Golpon H., Zardo P., Borlak J. (2021). miRNAs in lung cancer. A systematic review identifies predictive and prognostic miRNA candidates for precision medicine in lung cancer. Transl. Res..

[20] Armand-Labit V., Pradines A. (2017). Circulating cell-free microRNAs as clinical cancer biomarkers. Biomol. Concepts.

[21] Moretti F., D’Antona P., Finardi E., Barbetta M., Dominioni L., Poli A., Gini E., Noonan D.M., Imperatori A., Rotolo N. (2017). Systematic review and critique of circulating miRNAs as biomarkers of stage I-II non-small cell lung cancer. Oncotarget.

[22] Tiberio P., Callari M., Angeloni V., Daidone M.G., Appierto V. (2015). Challenges in using circulating miRNAs as cancer biomarkers. Biomed. Res. Int..

[23] Dinh T.-K.T., Fendler W., Chałubińska-Fendler J., Acharya S.S., O’Leary C., Deraska P.V., D’Andrea A.D., Chowdhury D., Kozono D. (2016). Circulating miR-29a and miR-150 correlate with delivered dose during thoracic radiation therapy for non-small cell lung cancer. Radiat. Oncol..

[24] Kwon J.J., Factora T.D., Dey S., Kota J. (2019). A Systematic Review of miR-29 in Cancer. Mol. Ther. Oncolytics.

[25] Wang F., Ren X., Zhang X. (2015). Role of microRNA-150 in solid tumors. Oncol. Lett..

[26] Lan F., Yu H., Hu M., Xia T., Yue X. (2015). miR-144-3p exerts anti-tumor effects in glioblastoma by targeting c-Met. J. Neurochem..

[27] Gao Z.Y., Liu H., Zhang Z. (2021). miR-144-3p increases radiosensibility of gastric cancer cells by targeting inhibition of ZEB1. Clin. Transl. Oncol..

[28] Wang P., Yang Z., Ye T., Shao F., Li J., Sun N., He J. (2020). lncTUG1/miR-144-3p affect the radiosensitivity of esophageal squamous cell carcinoma by competitively regulating c-MET. J. Exp. Clin. Cancer Res..

[29] Chen Y.-J., Guo Y.-N., Shi K., Huang H.-M., Huang S.-P., Xu W.-Q., Li Z.-Y., Wei K.-L., Gan T.-Q., Chen G. (2019). Down-regulation of microRNA-144-3p and its clinical value in non-small cell lung cancer: A comprehensive analysis based on microarray, miRNA-sequencing, and quantitative real-time PCR data. Respir. Res..

[30] Gao F., Liu P., Narayanan J., Yang M., Fish B.L., Liu Y., Liang M., Jacobs E.R., Medhora M. (2017). Changes in miRNA in the lung and whole blood after whole thorax irradiation in rats. Sci. Rep..

[31] Kooshkaki O., Rezaei Z., Rahmati M., Vahedi P., Derakhshani A., Brunetti O., Baghbanzadeh A., Mansoori B., Silvestris N., Baradaran B. (2020). MiR-144: A New Possible Therapeutic Target and Diagnostic/Prognostic Tool in Cancers. Int. J. Mol. Sci..

[32] Wang Q., Chen Y., Lu H., Wang H., Feng H., Xu J., Zhang B. (2020). Quercetin radiosensitizes non-small cell lung cancer cells through the regulation of miR-16-5p/WEE1 axis. IUBMB Life.

[33] Zhang W., Zhou F., Jiang D., Mao Y., Ye D. (2020). Association of the Expression Level of miR-16 with Prognosis of Solid Cancer Patients: A Meta-Analysis and Bioinformatic Analysis. Dis. Markers.

[34] Ghafouri-Fard S., Shoorei H., Branicki W., Taheri M. (2020). Non-coding RNA profile in lung cancer. Exp. Mol. Pathol..

[35] Yang L., Yang S., Ren C., Liu S., Zhang X., Sui A. (2022). Deciphering the roles of miR-16-5p in malignant solid tumors. Biomed. Pharmacother..

[36] Ghafouri-Fard S., Khoshbakht T., Hussen B.M., Abdullah S.T., Taheri M., Samadian M. (2022). A review on the role of mir-16-5p in the carcinogenesis. Cancer Cell Int..

[37] Małachowska B., Tomasik B., Stawiski K., Kulkarni S., Guha C., Chowdhury D., Fendler W. (2020). Circulating microRNAs as Biomarkers of Radiation Exposure: A Systematic Review and Meta-Analysis. Int. J. Radiat. Oncol. Biol. Phys..

[38] Faraldi M., Gomarasca M., Sansoni V., Perego S., Banfi G., Lombardi G. (2019). Normalization strategies differently affect circulating miRNA profile associated with the training status. Sci. Rep..

[39] Donati S., Ciuffi S., Brandi M.L. (2019). Human Circulating miRNAs Real-time qRT-PCR-based Analysis: An Overview of Endogenous Reference Genes Used for Data Normalization. Int. J. Mol. Sci..

[40] Lakkisto P., Dalgaard L.T., Belmonte T., Pinto-Sietsma S.-J., Devaux Y., de Gonzalo-Calvo D. (2023). Development of circulating microRNA-based biomarkers for medical decision-making: A friendly reminder of what should NOT be done. Crit. Rev. Clin. Lab. Sci..

[41] Hindson C.M., Chevillet J.R., Briggs H.A., Gallichotte E.N., Ruf I.K., Hindson B.J., Vessella R.L., Tewari M. (2013). Absolute quantification by droplet digital PCR versus analog real-time PCR. Nat. Methods.

[42] Campomenosi P., Gini E., Noonan D.M., Poli A., D’Antona P., Rotolo N., Dominioni L., Imperatori A. (2016). A comparison between quantitative PCR and droplet digital PCR technologies for circulating microRNA quantification in human lung cancer. BMC Biotechnol..

[43] Precazzini F., Detassis S., Imperatori A.S., Denti M.A., Campomenosi P. (2021). Measurements Methods for the Development of MicroRNA-Based Tests for Cancer Diagnosis. Int. J. Mol. Sci..

[44] Ke Y., Zhao W., Xiong J., Cao R. (2013). Downregulation of miR-16 promotes growth and motility by targeting HDGF in non-small cell lung cancer cells. FEBS Lett..

[45] Guo J., Yang Y.U.N., Zhao W.E.I., Yan Z., Yang X.I.A., Yan Y., Hao R., Hu J., Jiao F.E.I. (2021). MiR-16-5p plays an inhibitory role in human non-small cell lung cancer through Fermitin family member 2. Biocell.

[46] Li Y., Wang Z., Li Y., Jing R. (2017). MicroRNA-29a functions as a potential tumor suppressor through directly targeting CDC42 in non-small cell lung cancer. Oncol. Lett..

[47] Liu X., Lv X., Yang Q., Jin H., Zhou W., Fan Q. (2018). MicroRNA-29a Functions as a Tumor Suppressor and Increases Cisplatin Sensitivity by Targeting NRAS in Lung Cancer. Technol. Cancer Res. Treat..

[48] Zhang K., Han X., Hu W., Su C., He B. (2022). miR-29a-3p inhibits the malignant characteristics of non-small cell lung cancer cells by reducing the activity of the Wnt/β-catenin signaling pathway. Oncol. Lett..

[49] Pan H.-L., Wen Z.-S., Huang Y.-C., Cheng X., Wang G.-Z., Zhou Y.-C., Wang Z.-Y., Guo Y.-Q., Cao Y., Zhou G.-B. (2015). Down-regulation of microRNA-144 in air pollution-related lung cancer. Sci. Rep..

[50] Fang G., Zhang C., Liu Z., Peng Z., Tang M., Xue Q. (2022). MiR-144-3p inhibits the proliferation and metastasis of lung cancer A549 cells via targeting HGF. J. Cardiothorac. Surg..

[51] Dai F.-Q., Li C.-R., Fan X.-Q., Tan L., Wang R.-T., Jin H. (2019). miR-150-5p Inhibits Non-Small-Cell Lung Cancer Metastasis and Recurrence by Targeting HMGA2 and β-Catenin Signaling. Mol. Ther. Nucleic Acids.

[52] Grodzka A., Knopik-Skrocka A., Kowalska K., Kurzawa P., Krzyzaniak M., Stencel K., Bryl M. (2023). Molecular alterations of driver genes in non-small cell lung cancer: From diagnostics to targeted therapy. EXCLI J..

[53] Zhu X., Kudo M., Huang X., Sui H., Tian H., Croce C.M., Cui R. (2021). Frontiers of MicroRNA Signature in Non-small Cell Lung Cancer. Front. Cell Dev. Biol..

[54] Wang W.-T., Han C., Sun Y.-M., Chen T.-Q., Chen Y.-Q. (2019). Noncoding RNAs in cancer therapy resistance and targeted drug development. J. Hematol. Oncol..

[55] Long L., Zhang X., Bai J., Li Y., Wang X., Zhou Y. (2019). Tissue-specific and exosomal miRNAs in lung cancer radiotherapy: From regulatory mechanisms to clinical implications. Cancer Manag. Res..

[56] Yan H., Tang S., Tang S., Zhang J., Guo H., Qin C., Hu H., Zhong C., Yang L., Zhu Y. (2022). miRNAs in anti-cancer drug resistance of non-small cell lung cancer: Recent advances and future potential. Front. Pharmacol..

[57] Yang H., Liu Y., Chen L., Zhao J., Guo M., Zhao X., Wen Z., He Z., Chen C., Xu L. (2023). MiRNA-Based Therapies for Lung Cancer: Opportunities and Challenges?. Biomolecules.

[58] Wu K.-L., Tsai Y.-M., Lien C.-T., Kuo P.-L., Hung A.J.-Y. (2019). The Roles of MicroRNA in Lung Cancer. Int. J. Mol. Sci..

[59] MacLellan S.A., MacAulay C., Lam S., Garnis C. (2014). Pre-profiling factors influencing serum microRNA levels. BMC Clin. Pathol..

[60] Bache M., Rot S., Keßler J., Güttler A., Wichmann H., Greither T., Wach S., Taubert H., Söling A., Bilkenroth U. (2015). mRNA expression levels of hypoxia-induced and stem cell-associated genes in human glioblastoma. Oncol. Rep..

